# Assessment of Stone Columns as a Mitigation Technique of Liquefaction-Induced Effects during Italian Earthquakes (May 2012)

**DOI:** 10.1155/2014/216278

**Published:** 2014-01-27

**Authors:** Davide Forcellini, Angelo Marcello Tarantino

**Affiliations:** ^1^Department of Civil Engineering, University of San Marino, 44 Via Salita alla Rocca, 47890 San Marino, San Marino; ^2^Civil and Mechanical Engineering Department (DIMeC), University of Modena e Reggio Emilia, 905 Via Vignolese, 41100 Modena, Italy

## Abstract

Soil liquefaction has been observed worldwide during recent major earthquakes with induced effects responsible for much of the damage, disruption of function, and considerable replacement expenses for structures. The phenomenon has not been documented in recent time with such damage in Italian context before the recent Emilia-Romagna Earthquake (May 2012). The main lateral spreading and vertical deformations affected the stability of many buildings and impacted social life inducing valuable lessons on liquefaction risk assessment and remediation. This paper aims first of all to reproduce soil response to liquefaction-induced lateral effects and thus to evaluate stone column mitigation technique effectiveness by gradually increasing the extension of remediation, in order to achieve a satisfactory lower level of permanent deformations. The study is based on the use of a FE computational interface able to analyse the earthquake-induced three-dimensional pore pressure generation adopting one of the most credited nonlinear theories in order to assess realistically the displacements connected to lateral spreading.

## 1. Introduction

Liquefaction-induced deformations are one of the most dangerous collapse situations that can affect structures causing significant consequences such as damage, disruption of function, and considerable replacement expenses. When strong ground shaking occurs, this type of adverse response is commonplace as documented during the earthquakes of Niigata, Japan 1964 [1–4], Dagupan City, Philippines 1990, [5–7], Chi-Chi, Taiwan 1999 [8], Kocaeli, Turkey 1999 [9], and recent Tohoku earthquake, Japan 2011 [10–15].

During the recent Italian Emilia-Romagna Earthquakes (May 20 and May 29), liquefaction main effects were consequences of the first of these shocks (*M* = 5.9). The most significant phenomena of liquefaction have been observed in San Carlo settlement (located about 17 km from the epicentre), under the municipality of Sant'Agostino, near Ferrara. The main lateral and vertical deformations induced local and global instability to the buildings and roads closure. Many other typical postearthquake effects were observed such as uniform or differential settlements, sand boils, lateral spreading, soil raptures, water out coming, and many secondary effects. For more details, see Crespellani et al. [16].

Liquefaction mitigation measures generally consist of ground improvement, including removal and recompaction of low-density soils, removal of excess ground water, in situ ground densification, grouting, or surcharging, as described in California Geological Survey [17, Appendix F]. The choice of mitigation technique depends on the site conditions. In particular, the aim of this paper is to assess stone columns as a mitigation procedure to liquefaction-induced effects applied to Italian earthquakes.

## 2. Site Conditions and Remediation

The areas under investigation are classified on the national scale geological map as Holocene alluvial and fluvial-lacustrine soil deposits (http://www.pcn.minambiente.it/viewer/). The Southern part of the Ferrara Province is crossed by Reno River, the second most important river of the Emilia-Romagna Region after Po River. Reno River is an ancient river whose course varied over the plain throughout the centuries. Its waters often stagnated in a wide valley area between Bologna and Ferrara. Some interesting notes on the history of the Reno River can be found in Barbieri [18].

Although the biggest effects of soil liquefaction occur typically during strong earthquakes (Mw > 7.0) at susceptible sites close to the epicenter (e.g., M7.3 Charleston, USA 1886; M9.2 Alaska, USA 1964; M7.6 Niigata, Japan 1964; M7.6 Izmit, Turkey 1999), there have been cases where moderately strong earthquakes (e.g., M6.8 Kobe, Japan 1995; M6.3 Christchurch, New Zealand 2011) have produced widespread liquefaction. The May 20, 2012, M5.9 shock in Emilia Romagna, Italy, is one example of moderate earthquakes yielding extensive liquefaction-related phenomena.

The database of historical liquefaction in Italy Galli [19] demonstrates the existence of a relatively large number of weak to moderate earthquakes (MS > 4.2) producing liquefaction. The Italian territory is characterized by seismicity along the Apennine chain and Eastern Alps. Broad liquefaction-areas exist along the Adriatic, Tyrrhenian coasts, and the Po River alluvial plain. Field measurements were made to estimate the extension of the observed liquefaction phenomenon that was estimated in the first 15–20 m.

The superficial layer consists of paleobanks of alternation of sand (types S1 and S0). Below this strata (whose base is at about 13 m depth) a silt and clay layer (type A) with abundant organic fraction is located. The base of this layer corresponds to the Holocene and Pleistocene surface. The phreatic water table on June 8 and 18 was registered at around 6 to 8 m depth.

**Figure 1:**
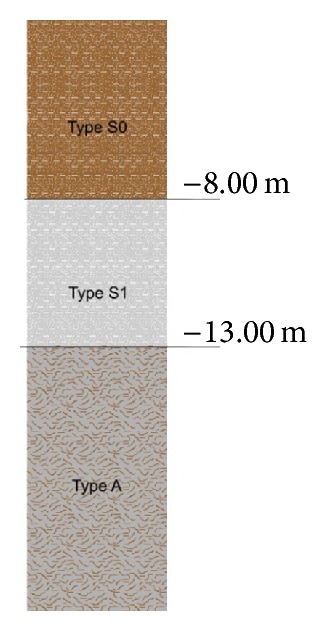
Vertical strata with superficial sand layers (type S0 and type S1) and silt and clay layer (type A).

In this paper, a 28 m geological model (Figure 1) has been built in order to be representative of several verticals taken in significant locations. The water level was prudentially considered at 0.00 m depth below surface.  Table 1 represents geotechnical parameters, such as density (*γ*), angle of internal friction (*φ*), shear wave velocity (*V*_*s*_), and permeability (*k*) for the several layers.

If compared to all the existing techniques, stone columns can be considered the less invasive and the most effective technique, because they combine beneficial effects of densification, reinforcement, and increased drainage (Priebe [20], Mitchell et al. [21], Japanese Geotechnical Society (JGS) [22], Thevanayagam et al. [23], Shenthan et al. [24]). In particular, gravel drains are a rather recent development compared with the more traditional soil densification approaches International Navigation Association (INA) [25]. Gravel drains technique was initially studied by Seed and Booker [26], and then, it has attracted the attention of many researchers such as Ishihara and Yamazaki [27]; Tokimatsu and Yoshimi [28]; Baez and Martin [29]; Boulanger et al. [30]; Brennan and Madabhushi [31]; and Elgamal et al. [32] as well as many practicing consultants as Nippon Kokan, Japan, and construction companies such as Konoike Construction in Japan and Hayward Baker in the United States (http://www.haywardbaker.com/). In 1985, the gravel drain technique received the Technical Development Award of the Civil Engineering Society of Japan (Saito et al. [33, 34]).

## 3. Computational Modeling

Current methodologies verify the risk for the soil to be subjected to liquefaction without a direct control on excess pore pressure. They only refer to few descriptive parameters based on historic knowledge or taken from geological and geotechnical recognitions and on behaviour analysis results of laboratory cycling test under controlled condition. These empirical methods are used only in preliminary studies allowing general considerations on liquefaction effects and deformation in qualitative terms (for more details, see Forcellini et al. [35]).

The aim of this paper is to realistically assess the entity of the displacements connected to lateral spreading assessing stone column remediation effectiveness. In this regard, study adopts credited nonlinear theories in order to take into account appropriate loading-unloading flow rules as to reproduce the observed strong dilation tendency and resulting increase in cyclic shear stiffness and strength (the “Cyclic Mobility” mechanism). For more details, see Yang and Elgamal [36], Yang et al. [37], and Elgamal et al. [38].

Even if based on these theories, the models have the main advantage to be built up with the most common-used geotechnical parameter. The other parameters are connected with the liquefaction mechanism and they can be obtained by assigned values calibrated on a big variety of realistic cases. In this regard, two different models for cohesionless and for cohesive soils were considered. The first model for cohesionless materials is developed within the framework of multi-yield-surface plasticity (Prevost [39]), focusing on controlling the magnitude of cycle-by-cycle permanent shear strain accumulation (Parra [40]; Yang [41]; Yang et al. [37]) by specifying an appropriate non-associative flow rule (Prevost [39]; Dafalias [42]; Bousshine et al. [43]; Nemat-Nasser and Zhang [44]; Radi et al. [45]). In particular, the deviatoric component of the flow rule is associative, while nonassociativity is restricted to the volumetric component only, as described in detail in Elgamal et al. [38]. Clay material is modelled as a nonlinear hysteretic material with a Von Mises multisurface Iwan [7] and Mróz [15] kinematic plasticity model, focusing on reproduction of the soil hysteretic elastoplastic shear response (including permanent deformation). The adopted parameters (Tables 2 and 3) were calibrated through an identification analysis taking into account nonlinear liquefaction-induced behaviors as specified in OpenSees PL manual Lu et al. [48]. More details on the calibration analysis are shown in Elgamal et al. [32], Forcellini and Tarantino [49], and Forcellini et al. [35].

The presented simulations were conducted using the open-source computational interface OPENSEES PL implemented in OpenSees Yang and Elgamal [36], Yang et al. [37], Yang [41], Mazzoni et al. [50]. It consists of a analysis framework for saturated soil response as a two-phase material following the *u*-*p* (where *u* is displacement of the soil skeleton and *p* is pore pressure) formulation of Chan [51] and Zienkiewicz et al. [52]. The soil domain is represented by 20-8 node, effective stress fully coupled (solid-fluid) brick elements Lu et al. [48] built up with 20 nodes describing the solid translational degrees of freedom and the eight-corner nodes for the fluid pressure Lu et al. [48]. In particular, OpenSees PL used in this study, originally calibrated for pile analyses, was modified in order to take into account stone columns behaviour (see also Elgamal et al. [32]). The interface simplifies the 3D spatial soil domain, boundary conditions, and input seismic excitation definition with convenient postprocessing and graphical visualization of the analysis results including the deformed ground response time histories (see Lu [53]). The ability to simulate the real wave propagation adopting realistic boundaries is of particular importance and significance in order to realistically reproduce the above scenarios.

**Figure 2:**
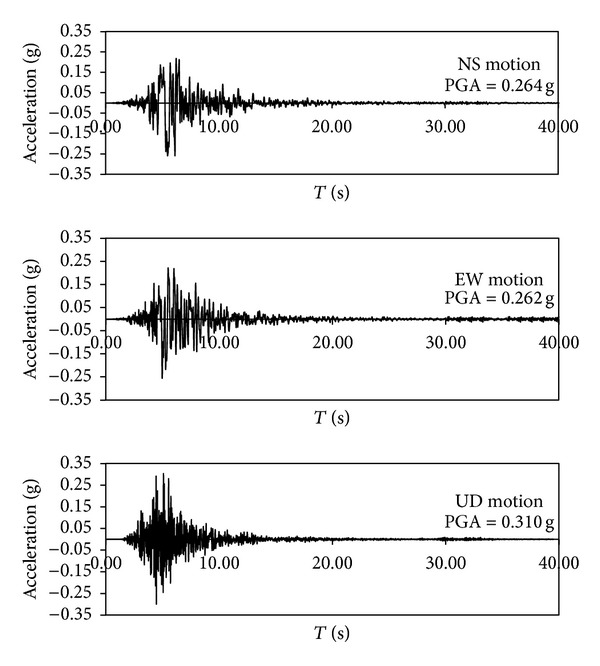
Recordings at Mirandola Station (MRN): NS, EW, and UD components [http://www.ingv.it/].

**Figure 3:**
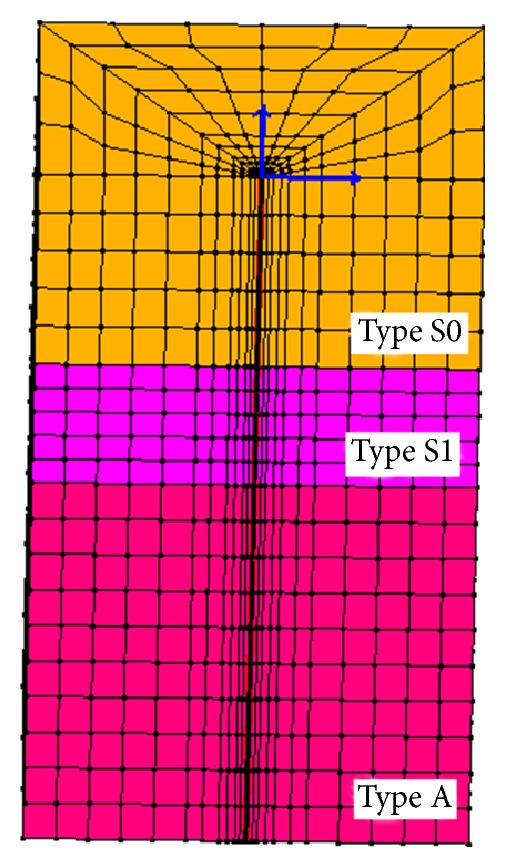
Free-field 3D model.

Recordings were taken from Mirandola (MRN) station, the closest station (about 13.4 km from the epicentre of May 20, 2012, shock).  Figure 2 shows the acceleration time histories of the North-South (NS), East-West (EW), and the vertical (UD) components, respectively, for each component recorded at MRN station. Two 3D models were considered. The first consists of a 3D 20 × 20 m, 28 m high model (Figure 3), representing the free field conditions and modeled with periodic boundary conditions on account of symmetry (at any spatial location displacement degrees of freedom of the left and right boundary nodes were tied together both longitudinally and vertically using the penalty method). Thus, the base and lateral boundaries were modeled to be impervious, as to represent a small section of a presumably infinite (or at least very large) soil domain by allowing the energy imparted by the seismic event to be removed from the site itself. For more details, see Law and Lam [54], Elgamal et al. [32], Forcellini and Tarantino [49], and Forcellini et al. [35]. The paper adopts a 308 elements mesh, assessed as the most representative compromise from a numerical time consuming calibration where several meshes (up to 3028 elements) were analysed and compared.

**Figure 4:**
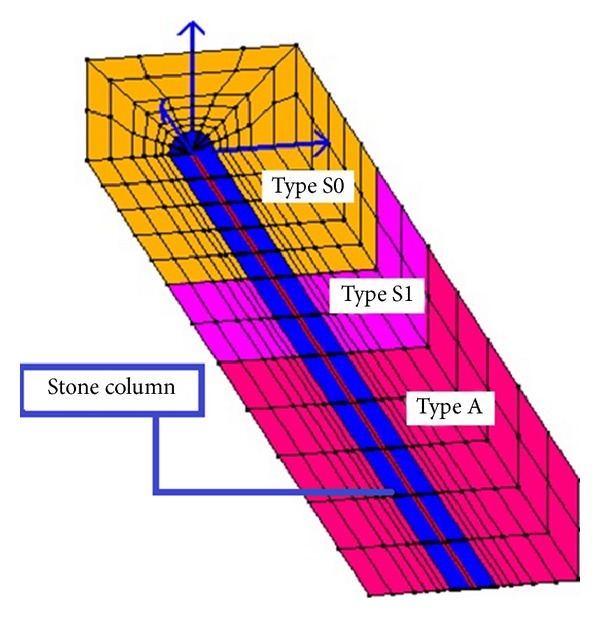
The 3D mitigation model.

The second model consists of a half mesh (Figure 4) simulating a representative cell within a large remediated ground zone. In particular, periodic boundaries offer an effective approach for conducting 3D analyses adopting the symmetry as to investigate a representative remediated “cell,” as shown in Law and Lam [54] and Elgamal et al. [32]. The paper adopts a 624 elements mesh, assessed as the most representative compromise from a numerical time consuming calibration where several meshes (up to 7080 elements) were analysed and compared.

## 4. Free Field Response

**Figure 5:**
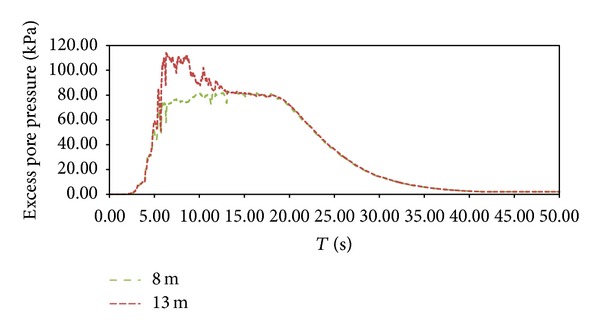
Excess pore pressure at 13.00 m and 8.00 m depth.

In this section free field results are shown in terms of excess pore pressure, longitudinal displacements time histories, and entire mesh deformation. In particular, Figure 5 shows that excess pore pressure reached around 120 kPa as the pick values at the base of S1 stratum (13 m) but started to rapidly decrease after about 15 s at the same level as 8 m and then is fully dissipated at 40 s. The results show that the pick value of pore pressure concentrates between 8.00 m and 13.00 m, meaning that S1 sand lent stratum results to be the principal cause of liquefaction-induced effects.

**Figure 6:**
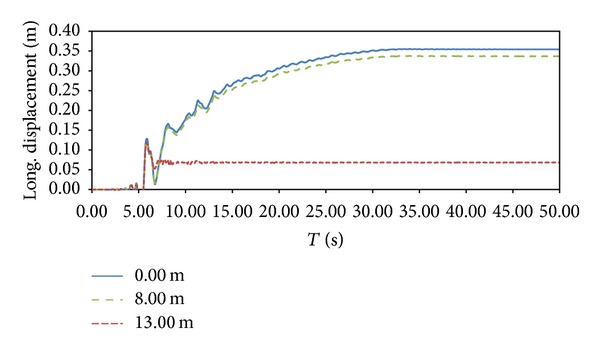
Longitudinal displacement at 13.00 m, 8.00 m, and 0.00 m depth.

**Figure 7:**
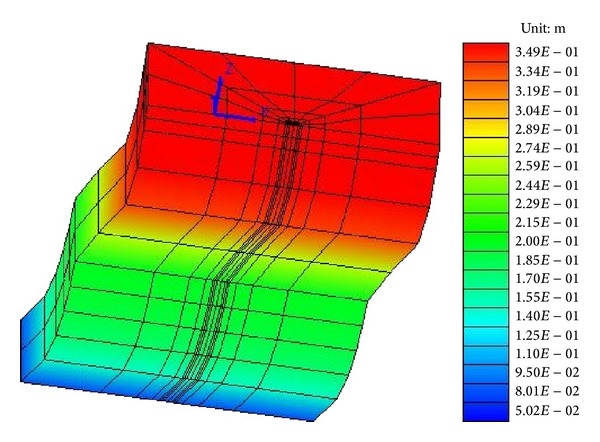
Deformed mesh at the end of the motion, scale 1:10.

This result is confirmed taking into consideration lateral displacements (Figure 6). At 8.00 m and 0.00 m depth displacements, no big difference in maximum values (around 32–35 cm) can be seen, while at 13.00 m depth, the final value is around 6 cm. This enforces the role of sand S1 layer in liquefaction-induced lateral spreading generation, since the lateral spreading is totally due to the layers between 8.00 m and 13.00 m. The entire mesh deformation (Figure 7) at the end of the motions registers such behaviour.

Another important consideration is the values of the modeled permanent displacement at the surface (around 35 cm), that is comparable to those measured during the reconnaissance in San Carlo free field conditions as shown in Crespellani et al. [16].

## 5. Assessment of Stone Columns Technique 

In this section stone column (SC) effectiveness in reducing the extent of liquefaction-induced lateral deformation is assessed taking into account several remediation cases. SC was represented by dense sand with gravel permeability of *k* = 0.01 m/s (for more details, see Table 4). In particular, the study assesses the remediation technique taking into account the area replacement ratio *A*_rr_, conventionally defined [32] as SC area (*A*_*r*_) to the tributary area *A*:

(1) $$\begin{array}{c} A_{\text{rr}}=\frac{A_{r}}{A}=\frac{\pi \cdot d^{2}}{S^{2}}, \end{array}$$

where *d* is stone column diameter and *S* is spacing between stone columns centers.

In order to take into account stone columns effectiveness, a parametric study varying *A*_rr_ from 0.5% to 20% as shown in Table 5 was performed.

**Figure 8:**
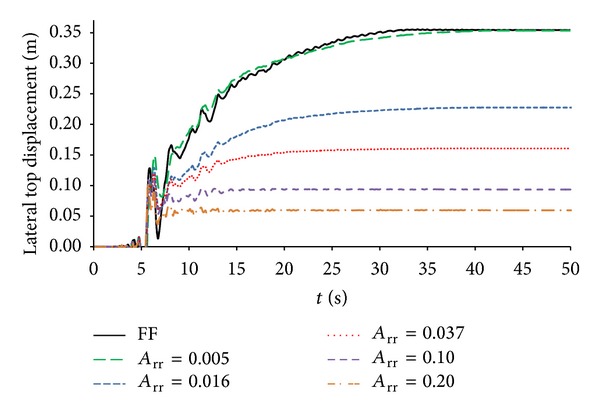
Lateral displacement at surface (0.00 m) time histories.

Figure 8 compares longitudinal displacements time histories at surface (0.00 m). In particular, it can be seen that free field value (35.4 cm) is very close with that of *A*_rr_ = 0.005 (35.0 cm), verifying the effectiveness of mitigation model with free field mesh: the more the replacement ratio increases, the more the lateral top displacement is reduced.

**Figure 9:**
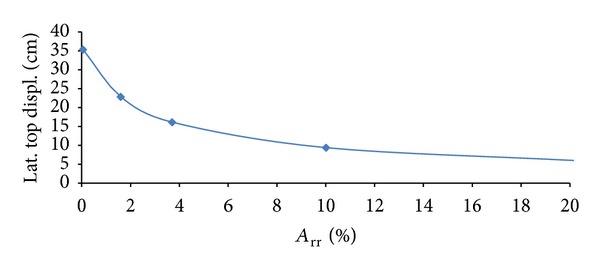
*A*_rr_-longitudinal top displacement assessment.

Figure 9 can be used to assess the best stone columns replacement ratio value (and consequently its remediation cost) compared with the required goal and performance to be obtained (such as minimizing lateral displacement). For example, with *A*_rr_ = 0.20, the lateral top displacement is around 6.0 cm that can be considered a suitable value for safety conditions.

**Figure 10:**
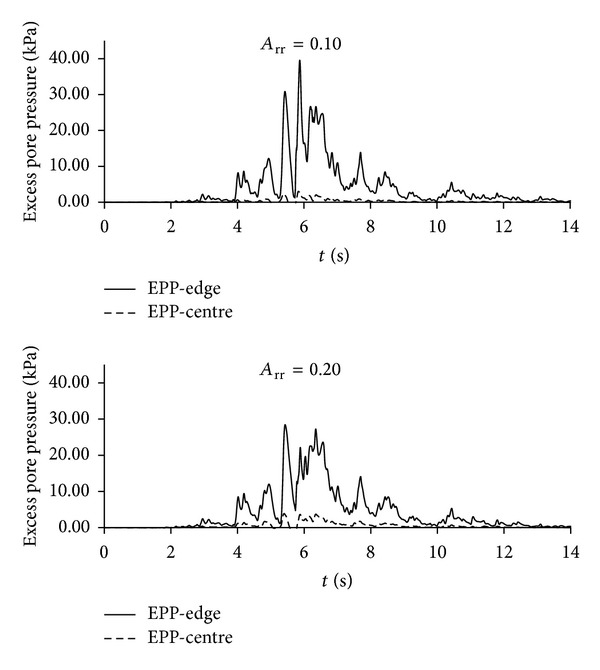
Excess pore-pressure time histories at the center and at the edge for *A*_rr_ = 0.10 and *A*_rr_ = 0.20.

Figure 10 shows excess pore-pressure time histories at the SC center compared with the far corner of the employed soil mesh (furthest location away from the SC) for *A*_rr_ = 0.10 and *A*_rr_ = 0.20 models. This helps to assess SC important role in reducing the extent of excess pore-pressure build-up. In particular, while in free field model the excess pore pressure reaches the highest level (around 120 kPa) after 10.00 sec and then it dissipates (Figure 5), in SC models, there is no significant pore-pressure generation within the SC zone. Therefore, the associated drastic reduction in pore pressure is shown to be an important factor in keeping deformations to a potentially tolerable level.

## 6. Conclusions

The paper presents computational modelling, free field response, and stone columns remediation assessment. A parametric study was conducted to assess the effectiveness of SC mitigation technique by gradually increasing the extension of remediation, in order to achieve a satisfactory lower level of permanent deformation. The analyses are aimed to numerically reproduce Italian Emilia-Romagna Earthquakes (May 2012) allowing several considerations.

First of all, free field response underlines the vulnerability of such submerged 3D system in terms of pore-pressure generations and lateral spreading values. Recordings from Mirandola (MRN) station induces typical postearthquake lateral spreading that is confirmed in this study. In particular, results verify the role of sand S1 layer in liquefaction-induced lateral spreading generation.

On the second hand, stone column remediation was found to be effective in reducing the sand stratum lateral deformation taking into consideration area replacement ratio (*A*_rr_) parameter. In particular, the response helps to assess the most suitable stone columns replacement ratio value (and consequently its remediation cost) compared with the required goal to minimize lateral displacements. Therefore, mitigation effectiveness and dimensioning design depend on the required performance to be provided in terms of safety level.

In this regard, this study can quantify soil performance to liquefaction-induced effects using metrics that are of immediate use for both preearthquake and postearthquake risk assessment analyses. This kind of response can become very powerful if applied to structures in soil-structure interaction studies. This will be object of further work.

## Figures and Tables

**Table 1:** Ground parameters for each layer.

	S0	S1	A
Mass density (kN/m^3^)	19.0	20.0	19.0
Internal angle of friction (°)	33.0	33.0	—
Shear wave velocity (m/s)	200	200	400
Permability (m/s)	10^−7^	10^−3^	—

**Table 2:** Characteristics adopted in the study for S0 and S1 strata.

	S0 (0–8 m)	S1 (8–13 m)
Mass density (kN/m^3^)	19.0	20.0
*G*_*r*_ low-strain shear modulus (MPa) at 80 kPa mean effective confinement	70	76
*B*_*r*_ low-strain bulk modulus (MPa) at 80 kPa mean effective confinement	180	200
Angle of internal friction (°)	33.0	33.0
Phase transformation angle (°)	26.5	27.0
Contraction parameter *c*_1_	0.3	0.3
Dilation parameter *d*_1_	0.4	0.4
Dilation parameter *d*_2_	2	2
Liquefaction parameter *l*_1_	10	10
Liquefaction parameter *l*_2_	0.01	0.01
Liquefaction parameter *l*_3_	3	3

**Table 3:** Characteristics adopted in the study for A stratum.

	A (13–28 m)
Mass density (kN/m^3^)	19.0
*G*_*r*_ low-strain shear modulus (MPa) at 80 kPa mean effective confinement	304
*B*_*r*_ low-strain bulk modulus (MPa) at 80 kPa mean effective confinement	1400
Apparent cohesion at zero effective confinement (kPa)	70

**Table 4:** Characteristics adopted in the study for SC.

	SC
Mass density (kN/m^3^)	19.0
*G*_*r*_ low-strain shear modulus (MPa) at 80 kPa mean effective confinement	135
*B*_*r*_ low-strain bulk modulus (MPa) at 80 kPa mean effective confinement	400
Angle of internal friction (°)	34.0
Phase transformation angle (°)	26.0
Contraction parameter *c*_1_	0.1
Dilation parameter *d*_1_	0.8
Dilation parameter *d*_2_	5
Permeability *k* (m/s)	0.01

**Table 5:** Stone columns models.

Name	*D* (m)	*A*_rr_ (%)	Displ (cm)
FF	—	—	35.40
*A*_rr_ = 0.005	0.10	0.05	35.30
*A*_rr_ = 0.16	0.60	1.60	22.80
*A*_rr_ = 0.37	1.00	3.70	16.10
*A*_rr_ = 0.10	2.00	10.00	9.30
*A*_rr_ = 0.20	3.70	20.00	6.00
